# Effects of Different River Crab *Eriocheir sinensis* Polyculture Practices on Bacterial, Fungal and Protist Communities in Pond Water

**DOI:** 10.3390/biom15010031

**Published:** 2024-12-30

**Authors:** Yun Bao, Bing Li, Rui Jia, Linjun Zhou, Yiran Hou, Jian Zhu

**Affiliations:** 1 College of Fisheries and Life Science, Shanghai Ocean University, Shanghai 201306, China; byshou@outlook.com; 2 Key Laboratory of Integrated Rice-Fish Farming Ecology, Ministry of Agriculture and Rural Affairs, Freshwater Fisheries Research Center, Chinese Academy of Fishery Sciences, Wuxi 214081, China; lib@ffrc.cn (B.L.); jiar@ffrc.cn (R.J.); zhoulinjun@ffrc.cn (L.Z.); 3 Wuxi Fisheries College, Nanjing Agricultural University, Wuxi 214081, China

**Keywords:** river crab polyculture, pond water, bacterial, fungal, protist communities, mandarin fish, silver carp

## Abstract

Microorganisms, including bacteria, fungi, and protists, are key drivers in aquatic ecosystems, maintaining ecological balance and normal material circulation, playing vital roles in ecosystem functions and biogeochemical processes. To evaluate the environmental impact of different river crab polyculture practices, we set up two different river crab (*Eriocheir sinensis*) polyculture practices: one where river crabs were cultured with mandarin fish (*Siniperca chuatsi*), silver carp (*Hypophthalmichthys molitrix*), and freshwater fish stone moroko (*Pseudorasbora parva*), and another where river crabs were cultured just with mandarin fish and silver carp. These two polyculture practices were referred to as PC and MC, respectively. We analyzed the water bacterial, fungal, and protist communities in the PC and MC groups using 16S, ITS, and 18S ribosomal RNA high-throughput sequencing. We found that the PC group obviously increased the diversity of microbial communities and altered their composition. The bacterial community held the narrowest habitat niche and exhibited the weakest environmental adaption compared to fungal and protist communities. The PC group altered the co-occurrence networks of bacteria, fungi, and protist, leading to more complex and stable communities of fungi and protist. Furthermore, the PC group shifted the assembly mechanism of the bacterial community from being predominantly deterministic to predominantly stochastic processes, with relatively minor impacts on the fungal and protist communities. Environmental factors, especially dissolved oxygen (DO), were significantly associated with the communities of bacteria, fungi, and protists, with DO being the major contributor to changes in the microbial communities. Our results suggest that the polyculture of river crab with mandarin fish, silver carp, and stone moroko was an effective and viable attempt, and it was superior in terms of microbial community diversity and stability.

## 1. Introduction

With the world’s population growing rapidly, aquaculture plays a crucial role in providing high-quality protein and addressing food security issues. Aquatic animals contribute over 20% to the global per capita supply of animal protein [1,2]. Polyculture is based on species compatibility and achieves complementarity between species through mutualism and trophic interactions, thereby generating higher profits and optimizing a more stable aquaculture environment [3,4]. The Chinese mitten crab (*Eriocheir sinensis*), also known as the river crab, has traditionally been welcomed as a high-valued aquatic product in China which is rich in nutrients and has a delicious flavor, making it an important component of the Chinese aquatic product market [5,6]. In China, the pond farming of river crabs typically adopts a polyculture production pattern. Aquatic grasses, filter-feeding fish, other crustaceans, and functional organisms work together to form cohesive ecological niches in river crab pond polyculture [7]. Traditional river crab polyculture practices in ponds mainly involve mandarin fish (*Siniperca chuatsi*), silver carp (*Hypophthalmichthys molitrix*), and freshwater prawn (*Macrobrachium nipponense*) [8,9,10,11]. Silver carp is a filter-feeding fish that primarily consumes phytoplankton and certain zooplankton, effectively controlling pond eutrophication and the excessive proliferation of plankton [12,13,14]. As an apex predator, mandarin fish co-cultured in crab pond can also effectively maintain a good culturing environment and support the survival of cultured species by suppressing toxic substances and eutrophication [15,16,17,18]. However, the rapid development of the river crab polyculture in ponds has led to an increasingly diverse selection of co-cultured species. Stone moroko *Pseudorasbora parva* is a small, omnivorous fish species that primarily feeds on algae, plankton, and detritus [19]. Due to its rapid growth and tender meat, the stone moroko is a high-quality aquatic product that is highly favored by consumers [20]. In recent years, stone moroko have increasingly been used in rice paddies and pond polyculture [21,22]. However, research on the polyculture of stone moroko and river crabs is still very limited. Hence, it is crucial to correctly assess the ecological functions and system sustainability of the various river crab-based polyculture practices.

Microorganisms, including bacteria, fungi, and protists, are key drivers in aquatic ecosystems, maintaining ecological balance and normal material circulation, and playing vital roles in ecosystem functions and biogeochemical processes [23,24,25,26,27,28,29]. Dynamic variations in bacterial communities indicate environmental changes or impacts, can reflect the ecological status of an ecosystem, and greatly influence the growth of aquatic animals [30,31,32]. Fungi possess enormous metabolic diversity and are essential components of organic matter transformation and energy cycling within aquatic ecosystems [33]. Prior research has found that fungi have positive effects on the mineralization of humic substances, which can greatly reduce the waste accumulation and improve the aquatic environment [34,35]. Protists usually act as primary producers, predators, and decomposers, maintaining ecological balance and playing a vital role in ecosystem construction and material transformation [36,37,38,39]. Additionally, microorganisms significantly impact aquatic animals in the aquaculture environment. The combined decomposition of complex organic matter by bacteria, fungi, and protists can promote water quality, providing a stable and suitable environment for the growth of aquatic organisms [40,41,42]. Both bacteria and fungi play essential roles in the prevention and control of diseases in aquatic organisms. Probiotics reduce pathogenic microorganisms through competitive exclusion mechanisms, simultaneously promoting gut health and enhancing the immunity of aquatic animals [43,44]. Certain fungi can suppress the growth of pathogens via secondary metabolites, offering natural alternatives to antibiotics in aquaculture systems [45].

Currently, water bacterial communities have been well studied in many ecosystems. Disturbances caused by feed input, aquaculture animals, and daily management can alter environmental parameters, thereby promoting further alterations in bacterial communities [18,46,47]. Composition variables in fungal communities have been observed to depend on aquatic animal disturbances and human activities, but the ecological roles of aquatic fungi remain poorly understood [48,49,50,51]. Protists in aquaculture environment are usually described as pathogenic factors of cultured animal or algae [52,53,54]. In recent years, people have started to focus on the interactions between water protist communities and aquaculture environment variables [55,56,57]. For example, protist communities in grass carp aquaculture ponds were found to be restructured through sediment resuspension caused by the fish’s movements [58]. Nevertheless, there is a lack of research on water protist communities in aquaculture environments [59]. The concurrent analysis of bacterial, fungal, and protist communities can provide a more comprehensive perspective on the aquaculture environment.

Therefore, we studied the bacterial, fungal, and protist communities in two different river crab polyculture ponds. The aims were to: (1) analyze variations in the microbial communities between two different river crab polyculture practices; (2) explore correlations between microbial community composition and environmental variables; (3) evaluate the performance of two river crab polyculture ecosystems based on the stability, adaptability, and diversity of microbial communities, among other factors. By assessing the aquaculture environment through microbial analysis, we aim to provide a theoretical and data foundation for sustainable river crab production.

## 2. Materials and Methods

### 2.1. Experimental Site and Sample Collection

This study was conducted at an aquaculture facility located in Changzhou (119.82° E, 31.99° N). Two standard aquaculture ponds, each measuring approximately 0.87 hectares, were selected and featured two types of river crab polyculture practices. One was the polyculture of river crab with mandarin fish (*S. chuatsi*) and silver carp (*H. molitrix*), represented as MC. The other one was the polyculture of river crab with mandarin fish, silver carp, and stone moroko (*P. parva*), represented as PC. All production parameters, including water exchanges, external inputs, daily management, etc., were strictly maintained consistently. The average weights of the stocked river carbs, mandarin fish, silver carp, and stone moroko were 15.63 g, 37.23 g, 682.22 g, and 9.21 g, respectively (Table S1). The stocking densities for the river carbs, mandarin fish, silver carp, and stone moroko were 21,000, 150, 900, and 800 ind·ha^−1^, respectively (Table S1). The farming practice began on March 15th and ended on October 15th. River crabs were fed once daily with a mix of commercial pellet feed, combined with fresh frozen trash fish and fermented corn in a ratio of 1:7:2. The commercial pellet feed was purchased from Xinbei Menghe Hongzhiyu Aquaculture Farm. The feeding amount was adjusted based on the weight of river crabs, approximately 3% of the total crab biomass in a pond. During the experimental trial, the water was changed every two weeks, with one-fifth of the pond water replaced each time.

Samples were collected during the peak period of river crab farming on 3 September 2023. In each pond, ten sampling sites were set up using a systematic sampling method to collect ten replicates. Water samples were gathered from each sampling site and filtered with 0.22 µm mixed cellulose ester membrane filter (Shxingya, Shanghai, China, Q/IEFJ01-1997). All membranes were collected for microbial sampling and stored at −80 °C for DNA extraction. The filtered water was immediately stored at −20 °C for analyses of ammonia (NH_4_^+^), nitrite (NO_2_^−^), nitrate (NO_3_^−^), total nitrogen (TN), phosphate (PO_4_^3−^), and total phosphorus (TP).

### 2.2. Measurement of Water Physiochemical Index

The pH and dissolved oxygen (DO) concentration were measured using a portable water quality analyzer (Hach, Loveland, CO, USA). The TP and TN concentrations were simultaneously determined using persulfate digestion, and ammonia content was determined employing Nessler’s Reagent Spectrophotometry method [60,61,62]. The levels of nitrate, nitrite, and phosphate were determined using Chinese national standard methods: nitrate was measured with the phenol disulfonic acid method, nitrite through the spectrophotometric method, and phosphate via the ammonium molybdate spectrophotometry method [63,64,65].

### 2.3. DNA Extraction, Sequencing, and Data Processing

Microbial DNA—including bacterial, fungal, and protist—was extracted from membranes utilizing the E.Z.N.A.^®^ Water DNA Kit (Omega Bio-tek, Norcross, GA, USA). Specific primers were designed and synthesized to amplify the ribosomal RNA genes of bacteria, fungi, and protists. The amplification was performed using an ABI GeneAmp^®^ 9700 PCR thermocycler (ABI, Oakland, CA, USA), and the amplified regions and primers are detailed in Table 1 [66,67,68]. Amplicons were isolated from 2% agarose gels and purified with the AxyPrep DNA Gel Extraction Kit (Axygen Biosciences, Union City, CA, USA). Furthermore, the NovaSeq library was created using the NEXTFLEX^®^ Rapid DNA-Seq Kit. Purified amplicons were pooled in equimolar amounts and subjected to paired-end sequencing on an Illumina NovaSeq PE250 platform (Illumina Inc, San Diego, CA, USA) at Biozeron Biotechnology Co., Ltd. (Shanghai, China).

We utilized FASTP (version 0.23.4) for quality control of the raw sequencing reads and employed FLASH (version 1.2.11) for assembly, featuring a minimum overlap of 10 bp and permitting a mismatch error rate of 2% [69,70]. Subsequently, the retained reads were dereplicated and examined using the Divisive Amplicon Denoising Algorithm 2 (DADA2) through Quantitative Insights into Microbial Ecology 2 (QIIME 2) to identify indel mutations and substitutions, prior to classifying them into amplicon sequence variants (ASVs) [71]. Paired reads were trimmed and filtered, allowing a maximum of two expected errors per read (maxEE ≤ 2). Bacterial, fungal, and protist ASVs were taxonomically classified and identified using the Silva (SSU132) database, the UNITE database, and the Protist Ribosomal Reference Database (PR2), respectively [72,73,74].

### 2.4. Statistical Analysis

To assess the alpha diversity of water bacterial, fungal, and protist communities, we computed the Shannon, Simpson, Chao1, and Pielou_J indices. We evaluated the difference in the composition of water bacterial, fungal, and protist communities between the PC and MC groups using Principal Coordinates Analysis (PCoA) grounded in weighted Bray–Curtis distances [75]. The habitat niche breadth and dispersal ability were calculated to evaluate the environmental adaption of microbial communities [76,77]. Microbes were divided into transient, intermittent, and persistent types, and further compared in terms of their numbers and abundance to evaluate the environmental sensitivity [78]. Correlations of the water physiochemical variables with microbial communities were analyzed by Mantel’s test using Bray–Curtis distance matrices with 999 permutations [79]. The relative contribution of water physiochemical variables on microbial communities was illustrated using aggregated boosted tree (ABT) analysis [80]. Further, co-occurrence patterns were established, founded on Spearman’s correlation matrices (Spearman *r* > 0.6 and *p*-value < 0.05) of 16S rRNA, ITS, and 18S rRNA sequencing data [81]. Additionally, a neutral community model (NCM) was used to assess the relative contributions of deterministic and stochastic processes to the assembly of microbial communities in pond water, and within this model, the value of “R2” indicates the model’s fit, with a higher “R2” value suggesting a greater influence of stochastic processes in community assembly [82,83,84,85].

## 3. Results

### 3.1. Microbial Community Diversity in Pond Water

**Figure 1:**
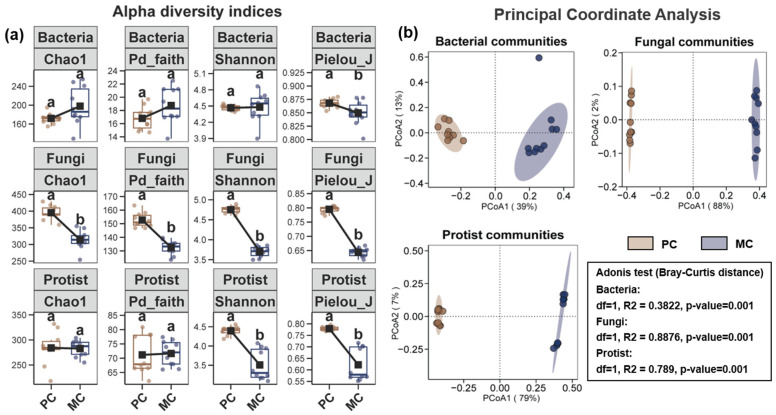
Variations in water microbial community diversity between the PC and MC groups. (**a**) Differences in the Chao1, Shannon, Pd_faith, and Pielou_J indices within water bacterial, fungal, and protist communities between the PC and MC groups. Distinct letters represent statistically significant differences in each index between the PC and MC groups (*p* < 0.05). (**b**) Principal coordinate analysis (PCoA) and Adonis test of water bacterial, fungal, and protist communities between the PC and MC groups.

Differences in the alpha and beta diversities of the microbial community within pond water are illustrated in Figure 1. For the bacterial community in pond water, the Pielou_J indices in the PC group was significantly higher than in the MC group (Figure 1a, *p* < 0.05). In contrast, the Chao1, Pd_faith, and Shannon indices of the water bacterial community did not show any remarkable differences between the two groups (Figure 1a, *p* > 0.05). The Chao1, Pd_Faith, Shannon, and Pielou_J indices of the fungal community in pond water were substantially greater in the PC group compared to the MC group (Figure 1a, *p* < 0.05). Similarly, for the protists community in the PC group, both the Shannon and Pielou_J indices were notably higher than those in the MC group (Figure 1a, *p* < 0.05). Moreover, the bacterial, fungal, and protist communities were clearly grouped between the PC and MC groups, as demonstrated by PCoA analysis and Adonis tests (Figure 1b, *p* < 0.05).

### 3.2. Microbial Community Composition in Pond Water

**Figure 2:**
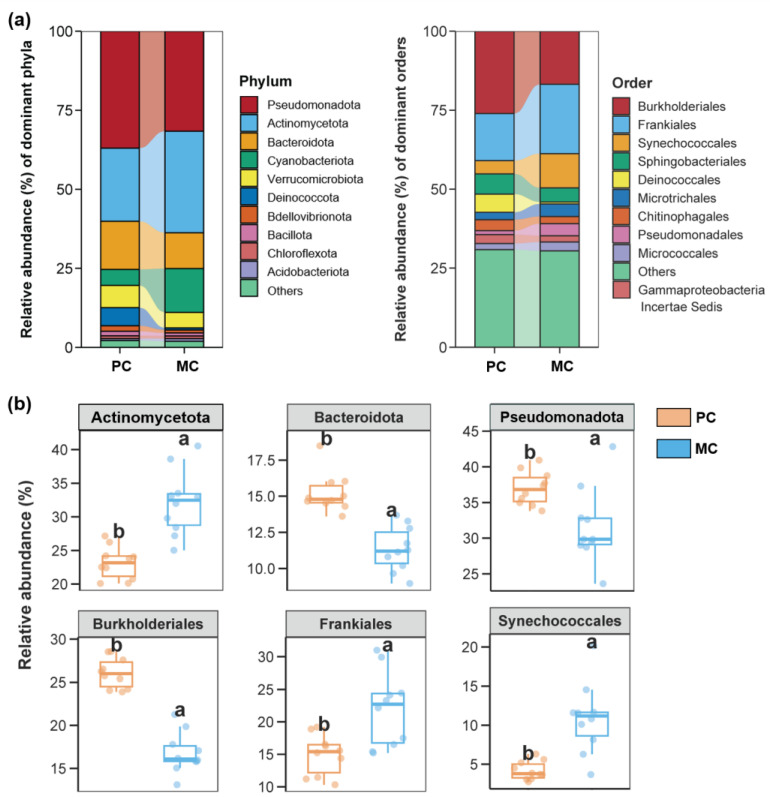
Bacterial communities in the pond water across the PC and MC groups. (**a**) The dominant phyla and orders (top 10 most abundant) of the bacterial communities in pond water across the PC and MC groups. (**b**) Differences in the relative abundance of the top 3 abundant bacterial phyla and orders between the PC and MC groups. Different lowercase letters above each box in the same subfigure represent significant differences between groups (*p* < 0.05).

**Figure 3:**
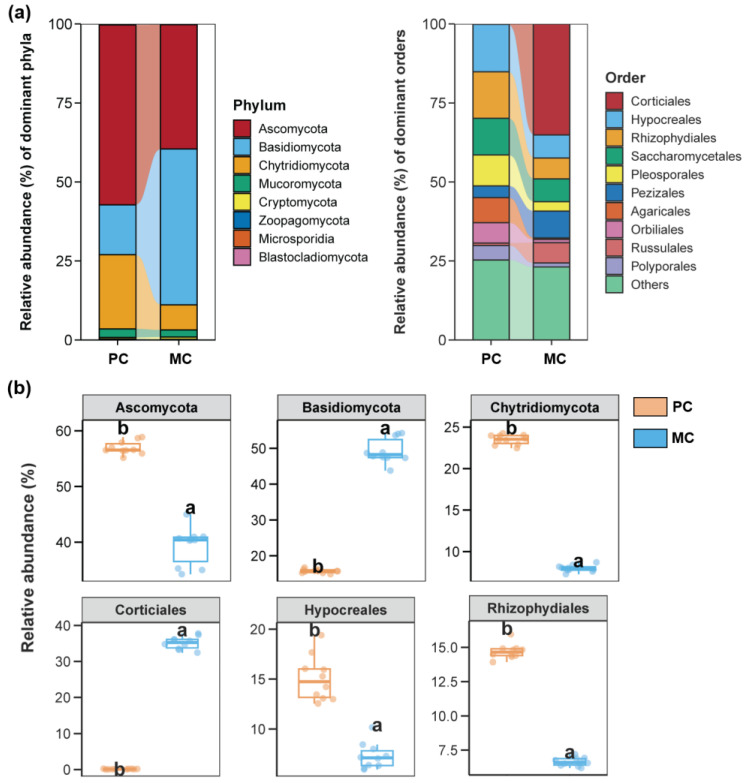
Fungal communities in the pond water across the PC and MC groups. (**a**) The dominant phyla and orders (top 10 most abundant) of the fungal communities in pond water across the PC and MC groups. (**b**) Differences in the relative abundance of the top 3 abundant fungal phyla and orders between the PC and MC groups. Different lowercase letters above each box in the same subfigure represent significant differences between groups (*p* < 0.05).

**Figure 4:**
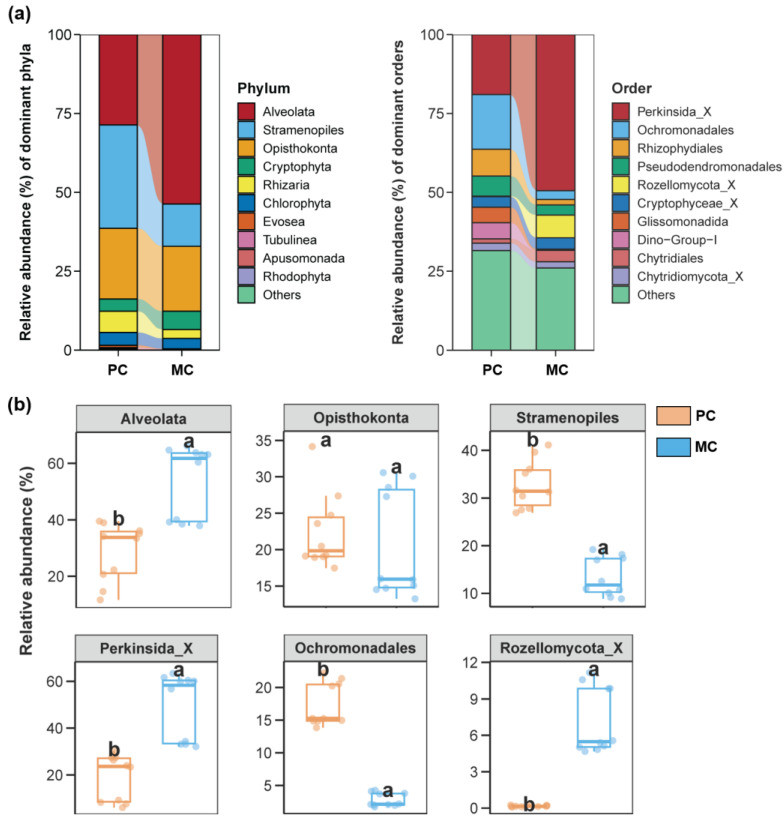
Protist communities in the pond water across the PC and MC groups. (**a**) The dominant phyla and orders (top 10 most abundant) of protist communities in pond water across the PC and MC groups. (**b**) Differences in the relative abundance of the top 3 abundant protist phyla and orders between the PC and MC groups. Different lowercase letters above each box in the same subfigure represent significant differences between groups (*p* < 0.05).

The composition of water bacterial, fungal, and protist communities exhibited substantial differences between the PC and MC groups (Figure 2, Figure 3 and Figure 4). In our study, the bacterial community at the phylum level was primarily composed of Pseudomonadota, Actinomycetota, and Bacteroidota, with Burkholderiales, Frankiales, and Synechococcales being the three dominant orders in the bacterial community (Figure 2b). In the PC group, Pseudomonadota, Bacteroidota, and Burkholderiales were remarkably more prevalent and matched up with the MC group (Figure 2b, *p* < 0.05). Conversely, the PC group exhibited a notably higher proportion of Actinomycetota, Frankiales, and Synechococcales in contrast to the MC group (Figure 2b, *p* < 0.05).

For the water fungal community, the top three most dominant phyla in terms of relative abundance were Ascomycota, Basidiomycota, and Chytridiomycota. The top three most abundant orders of fungi in the PC group were Hypocreales, Rhizophydiales, and Saccharomycetales, whereas Corticiales, Pezizales, and Saccharomycetales dominated the MC group (Figure 3a). In the PC group, Ascomycota, Chytridiomycota, Hypocreales, and Rhizophydiales held a substantially larger proportion in contrast with the MC group. (Figure 3b, *p* < 0.05). Conversely, Basidiomycota and Corticiales were obviously more abundant in the MC group than in the PC group (Figure 3b, *p* < 0.05).

For the water protist community, at the phylum level, Alveolate, Stramenopiles, and Opisthokonta were the most abundant in both groups. In the PC group, Perkinsida_X, Ochromonadales, and Rhizophydiales occupied the top three most abundant orders, whereas Perkinsida_X, Rozellomycota_X, and Cryptophyceae_X dominated in the MC group (Figure 4a). The PC group exhibited remarkably higher levels of Stramenopiles and Ochrominales compared to the MC group, whereas Alveolata, Perkinsida_X, and Rozellomycota_X were notably lower in the PC group against the MC group (Figure 4b, *p* < 0.05).

### 3.3. Environmental Adaption for the Pond Water Microbial Community

**Figure 5:**
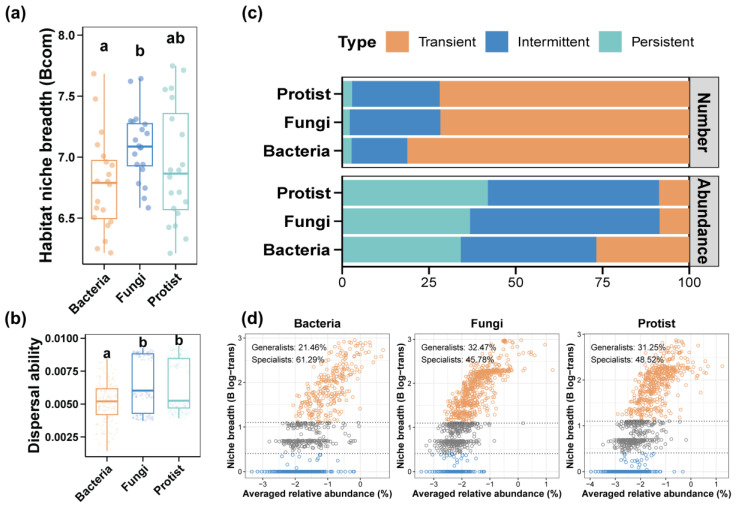
Environmental adaption of pond water microbial community. (**a**) Habitat niche breadth of bacterial, fungal, and protist community. (**b**) Dispersal ability of bacterial, fungal, and protist communities. (**c**) Proportion of transient, intermittent, and persistent microbial community calculated on number and abundance. (**d**) Proportion of microbial generalists and specialists in pond water. Different lowercase letters above each box in the same subfigure represent significant differences between groups (*p* < 0.05).

The habitat niche breadth of the fungal community was remarkably wider matched up with the bacterial community (Figure 5a, *p* < 0.05), and the protist community showed no significant difference compared to the bacterial and fungal communities (Figure 5a, *p* > 0.05). For whole samples, the dispersal ability of fungal and protist communities was significantly stronger than that of the bacterial community (*p* < 0.05), and no noticeable distinctions were found between the fungal and protist communities (Figure 5b, *p* > 0.05). The proportions of transient, intermittent, and persistent microbes were calculated based on the ASV numbers and relative abundances (Figure 5c). The proportions of the transient, intermittent, and persistent species for the bacterial, fungal, and protist communities were similar (Figure 5c). Notably, the bacterial community exhibited the highest proportion of transient species and the lowest proportion of intermittent and persistent species compared to the fungal and protist communities in pond water (Figure 5c). Furthermore, based on the habitat niche breadth, microbes were classified as generalists or specialists. The bacterial communities in the two ponds had the highest proportion of specialists (61.29%) compared to fungal (45.78%) and protist (48.52%) communities. Conversely, bacterial communities had the lowest proportion of generalists (21.46%) compared to fungal (32.47%) and protist (31.25%) communities (Figure 5d).

### 3.4. Assembly Processes Shaping the Pond Water Microbial Community

**Figure 6:**
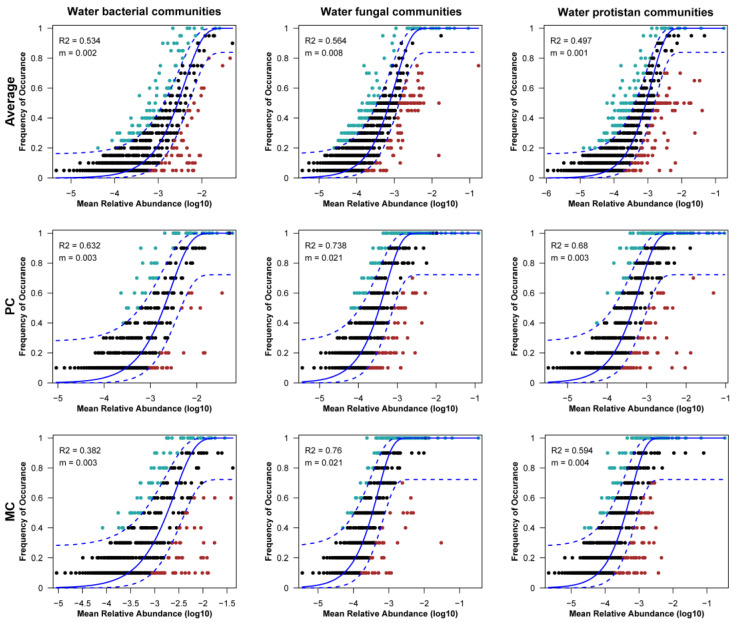
Ecological processes shaping the assembly of bacterial, fungal and protist communities in two river crab polyculture ponds. The neutral community model (NCM) of microbial communities in the PC and MC groups was illustrated, with solid lines representing the best fit of the model, and dashed lines indicating the 95% confidence interval around the predictions. The parameter “m” denotes the migration rate, and the “R^2^” value reflects the model’s goodness of fit.

According to the results of the neutral community model, stochastic processes had a greater impact on the water bacterial and fungal communities, with average R^2^ values of 0.534 and 0.564, respectively, across the two ponds (Figure 6). In contrast, water protist communities were less affected by stochastic processes, with an R^2^ value of 0.497. Furthermore, within the bacterial community assembly process in each pond, the PC group (R^2^ = 0.632) was more influenced by stochastic process than the MC group (R^2^ = 0.382). Fungal communities in both ponds were predominantly governed by stochastic processes, with R^2^ values of 0.738 for the PC group and 0.76 for the MC group. For the protist community, the PC group (R^2^ = 0.68) was more influenced by stochastic processes than the MC group (Figure 6, R^2^ = 0.594).

### 3.5. Co-Occurrence Network Patterns for the Pond Water Microbial Community

**Figure 7:**
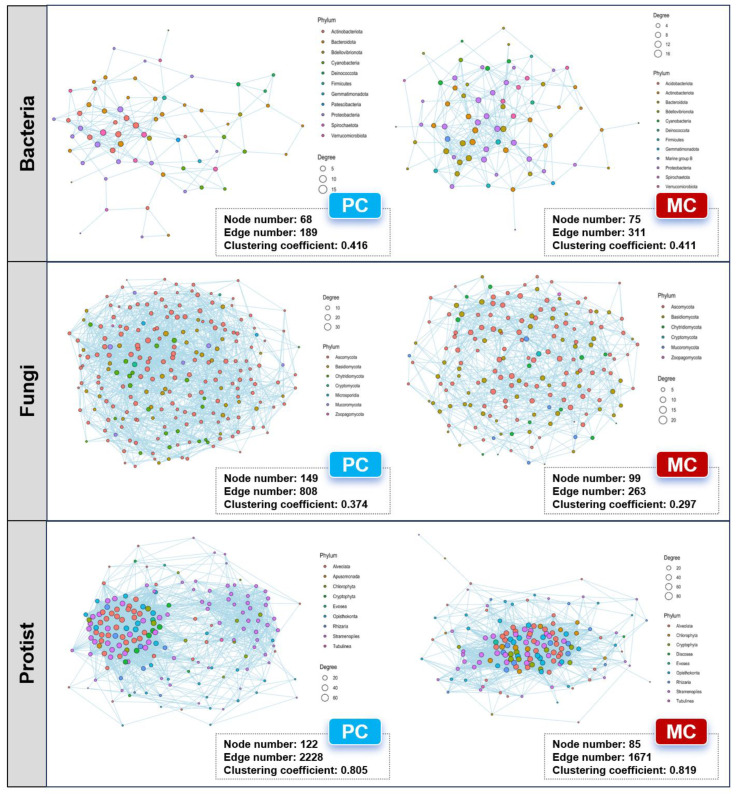
The co-occurrence networks for the bacterial, fungal, and protist communities in pond water across the PC and MC groups.

The co-occurrence networks of microbes in two ponds were constructed at the phylum level. As the results show, fungal and protist networks in the PC group were larger in size (in terms of edges and nodes) than those in the MC group. For fungal networks, the PC group contained 149 nodes and 808 edges, whereas the MC group had 99 nodes and 263 edges (Figure 7). In the protist networks, the PC group comprised 122 nodes and 2228 edges, while the MC group consisted of 85 nodes and 1671 edges. (Figure 7). For bacterial networks, no obvious distinctions were observed between the groups. The PC group had 68 nodes and 189 edges, while the MC group had 75 nodes and 311 edges (Figure 7). There were no notable variations in the clustering coefficient of bacterial and protist networks between different groups. However, the fungal network in the PC group had a higher clustering coefficient evaluated against the MC network, with values of 0.374 and 0.297, respectively (Figure 7).

### 3.6. Environmental Variables and Their Correlations with Pond Water Microbial Community

**Figure 8:**
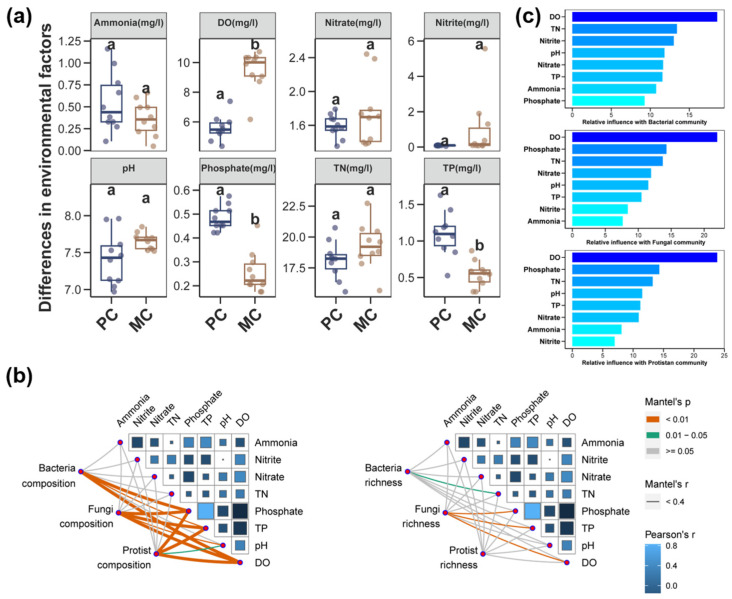
Environmental variables and their interaction with microbial community. (**a**) Difference in the ammonia, dissolved oxygen (DO), nitrate, nitrite, pH, phosphate, total nitrogen (TN), and total phosphorus (TP) concentrations in pond water between the PC and MC groups. Different lowercase letters indicate significant differences (*p* < 0.05). (**b**) Correlations of the water physiochemical variables with microbial composition and richness. (**c**) ABT analysis on the environmental physiochemical variable’s relative influence with bacterial, fungal, and protist communities.

Differences in environmental variables between the PC and MC groups are presented in Figure 8a. There were no considerable differences in ammonia, TN, nitrite, nitrate, and pH levels in pond water between the PC and MC groups (Figure 8a, *p* > 0.05). However, the concentrations of phosphate and TP were obviously higher in the PC group compared with the MC group (Figure 8a, *p* < 0.01). Conversely, the DO level was notably higher in the MC group than in the PC group (Figure 8a, *p* < 0.01).

According to Mantel’s test, the concentrations of phosphate, TP, and the DO showed significant correlations with the composition of bacterial, fungal, and protist communities (Figure 8b, *p* < 0.01). pH levels were strongly correlated with the composition of fungal and protist communities. (Figure 8b, *p* < 0.05). TN exhibited a significant relationship with the richness of the bacterial community (Figure 8b, *p* < 0.05), while phosphate, TP, and DO were strongly correlated with richness of fungal community (Figure 8b, *p* < 0.01). According to the ABT analysis (Figure 8c), the contribution of environmental factors affecting bacteria were DO, TN, and nitrite in descending order. For fungal and protist communities, the key environmental factors, listed in descending order of influence, were DO, phosphate, and TN.

## 4. Discussion

### 4.1. Different River Crab Polyculture Practices Impacted Microbial Diversity and Composition in Pond Water

In our study, the alterations in microbial α-diversity indices including Pielou_J and Shannon suggest that the PC group promoted the evenness of the bacterial community and enhanced the diversity of fungal and protist communities [86,87,88]. The dominant bacterial and fungal phyla in both the PC and MC groups included Pseudomonadota, Actinomycetota, Bacteroidota, Ascomycota, Basidiomycota, and Chytridiomycota, which are consistent with those typically observed in freshwater aquaculture environments [89,90,91,92,93,94]. For protist communities, the most dominant phyla were Alveolata, Stramenopiles, and Opisthokonta, resembling those found in natural aquatic systems such as lakes, rivers, and streams [95,96,97].

Different river crab polyculture practices obviously altered the microbial community composition. Specifically, the PC group exhibited a marked increase in the proportion of Pseudomonadota and Bacteroidota, which are critical for breaking down organic materials and accelerating nutrient cycling [98,99,100]. Likewise, in the PC group, the relative abundances of Ascomycota and Chytridiomycota were significantly higher than those in the MC group. These fungi are known for their ability to decompose complex organic matters, such as lignin and cellulose, thereby contributing to biodegradation processes [101,102,103,104]. For protist communities, the PC group displayed a higher abundance of Stramenopiles contrasted with the MC group. Stramenopiles act as both consumers and producers in aquatic environments, playing crucial roles in carbon and mineral cycling [105]. These notable differences in microbial community composition under different river crab polyculture practices highlight the profound effects on material cycling mediated by microbes. Previous studies corroborate that polyculture practices influence microbial communities through interspecific interactions and bioturbation [89,106]. Consequently, the observed differences in microbial community composition in our study could be attributed to ecological changes induced by the introduction of stone moroko.

### 4.2. Different River Crab Polyculture Practices Impacted Microbial Assembly Processes in Pond Water

Deterministic factors and stochastic factors represent the two main processes in the assembly of microbial communities, with their relative impacts varying across ecosystems [18,58,107,108]. Deterministic processes shape microbial communities through biological factors, such as species interactions, as well as abiotic factors, including environmental conditions [109]. In contrast, stochastic processes are primarily driven by dispersal mechanisms (migration and drift, etc.) and stochastic events (birth, death, and extinction, etc.) [83].

In our study, the NCM highlighted a clear distinction of bacterial community assembly processes between the PC (R^2^ = 0.632) and MC (R^2^ = 0.382) groups. The PC bacterial community assembly was predominantly influenced by stochastic processes. In contrast, the assembly of the MC bacterial community was primarily governed by deterministic processes. Previous studies confirm that environmental variables (DO, TN, TP, etc.) can modulate the relative importance of deterministic and stochastic processes in bacterial community assembly [85,110,111]. Furthermore, species interactions driven by different polyculture practices also influence the bacterial community assembly process [18]. This suggests that the bacterial communities in our study were significantly impacted by environmental variables associated with varying polyculture practices. For fungal and protist communities, no notable differences were observed between the PC and MC groups, with stochastic processes dominating the assembly of these communities.

### 4.3. Different River Crab Polyculture Practices Impacted Microbial Co-Occurrence Network and Environmental Adaption

A co-occurrence network can elucidate the intricate interactions among microorganisms [112]. The nodes and edges represent the complexity of the network [113]. A more complex and closely connected network will exhibit greater stability [114,115,116]. In our study, the fungal and protist networks in the PC group exhibited significantly more interactions compared to the MC group, suggesting more intricate and more stable fungal and protist communities in the PC group. The notably increased diversity induced in our study also confirmed the more stable fungal and protist communities in the PC group [117,118]. Conversely, the bacterial networks were slightly influenced by the different polyculture practices.

We further analyzed the environment’s adaption and sensitivity to provide an ecological insight into the stability of microbial communities. Based on the habitat niche breadth, microbial communities can be divided into specialists and generalists [119,120]. Specialists, rather than generalists, are more vulnerable and susceptible to environmental change, and high proportion of specialists means a strong environmental sensitivity [121]. The proportion of specialists in the bacterial community was much higher compared to fungal and protist communities. And the proportion of generalists in the bacterial community was the lowest among bacterial, fungal, and protist communities. We also calculated the proportion of transient, intermittent, and persistent microbial communities. The higher proportion of transient and the lower proportion of persistent communities represent the stronger environmental sensitivity [78]. Our results indicate that the bacterial community was more sensitive than the fungal and protist communities. In addition, the bacterial community holds the narrowest habitat niche, compared to the fungal and protist communities; a narrower habitat niche breadth represents a weaker metabolic plasticity, which indicates the weak environmental adaption of the bacterial community [122].

### 4.4. Impact of Environmental Variables to Bacterial, Fungal, and Protist Communities

In our study, both ABT and Mantel’s test showed a strong correlation between microbial communities and DO concentration. It is well-known that DO provide the oxygen necessary for microbial growth, reproduction, and metabolism. Previous studies have demonstrated that variations in DO concentration can affect the activity of bacterial enzymes and the structure of bacterial communities [110,123,124,125]. For fungi, a low-oxygen environment can reduce diversity, impair their decomposition abilities, and alter community composition [126,127,128]. Numerous studies have also reported a strong correlation between DO concentration and protist communities [129,130,131]. Additionally, Mantel’s test revealed a strong correlation between the concentrations of phosphate and total phosphorus with the compositions of bacterial, fungal, and protist communities. The concentration of total nitrogen was primarily associated with the richness of bacterial communities. Nitrogen and phosphorus, essential nutrients for microbial reproduction, can regulate the growth of microorganisms [132,133,134,135]. Previous research has identified significant correlations between total nitrogen concentration and bacterial community composition in both shrimp and grass carp aquaculture ponds [111,136]. Several studies have indicated that variations in phosphorus concentration can also alter the compositions of fungal and protist communities [58,137,138,139,140].

Therefore, in our study, changes in microbial communities, including composition, diversity, assembly processes, co-occurrence networks, and especially environmental stability, might be affected by variations in environmental factors, particularly those driven by DO.

## 5. Conclusions

In summary, different river crab polyculture practices notably affected the communities of bacteria, fungi, and protists. Compared to the MC group, the addition of stone moroko in the PC group substantially enhanced the diversity of bacterial, fungal, and protist communities and altered the composition of these communities. Among all samples, bacterial communities exhibited the narrowest habitat niche and the weakest adaptability to environmental changes relative to fungi and protist. The PC group modified the co-occurrence networks of bacteria, fungi, and protists, leading to more complex and stable communities of fungi and protists. Furthermore, the PC group shifted the assembly mechanism of the bacterial community from predominantly deterministic to predominantly stochastic processes, with relatively minor impacts on the fungal and protist communities. Environmental factors, particularly DO, were significantly associated with bacterial, fungal, and protist communities, with DO serving as the primary driver of changes in microbial communities. Our results suggest that the polyculture of river crab with mandarin fish, silver carp, and stone moroko was an effective and viable approach, and it was superior in terms of microbial community diversity and stability. Our study provides a theoretical and data foundation for the sustainable development of agriculture, especially aquaculture production patterns.

## Figures and Tables

**Table 1:** Amplification regions and primers for bacterial, fungal, and protist DNA.

Category	Amplification Region	Primer
Bacteria	16S rRNA V3-V4	338F (5′-ACTCCTACGGGAGGCAGCAG-3′)
		806R (5′-GGACTACHVGGGTWTCTAAT-3′)
Fungi	ITS1-ITS2	ITS1F (5′-CTTGGTCATTTAGAGGAAGTAA-3′)
		ITS2R (5′-GCTGCGTTCTTCATCGATGC-3′)
Protist	18S rRNA V4	TAReuk454FWD1 (5′-CCAGCASCYGCCGTAATTCC-3′)
		TAReUkREV3 (5′-ACTTTCGTTCTTTGATYRA-3′)

## Data Availability

The microbial (bacteria, fungi, and protist) data that support the findings of this study are openly available from the National Center for Biotechnology Information (NCBI) with the access numbers PRJNA1171740, PRJNA1171751, and PRJNA1171755, respectively.

## Supplementary Materials

The following supporting information can be downloaded at https://www.mdpi.com/article/10.3390/biom15010031/s1, Table S1: The cultured organisms and their corresponding stocking densities in the PC and MC groups.
