# Supplementary material for: Effects of Different River Crab Eriocheir sinensis Polyculture Practices on Bacterial, Fungal and Protist Communities in Pond Water
Source: Biomolecules. 2024 Dec 30;15(1):31. doi: 10.3390/biom15010031 (PMC11761872; doi:10.3390/biom15010031)
Supplement: Supplementary file 1 [file biomolecules-15-00031-s001.zip › biomolecules-3305202-supplementary.pdf]

Supplementary Materials

Table S1. The cultured organisms and their corresponding stocking densities in the PC and MC groups.

Treatment	Aquaculture Species	Item		Period
PC	*Eriocheir sinesis*	Body weight/g	15.63	March-October
		Density/ind·ha^{-1}	21000	
	Siniperca chuatsi	Body weight/g	37.23	May-October
		Density/ind·ha^{-1}	150	
	Hypophthalmichthys molitrix	Body weight/g	682.22	March-October
		Density/ind·ha^{-1}	900	
	Pseudorasbora parva	Body weight/g	9.21	June-October
		Density/ind·ha^{-1}	800	
MC	*Eriocheir sinesis*	Body weight/g	15.51	March-October
		Density/ind·ha^{-1}	21000	
	Siniperca chuatsi	Body weight/g	37.23	May-October
		Density/ind·ha^{-1}	150	
	Hypophthalmichthys molitrix	Body weight/g	682.22	March-October
		Density/ind·ha^{-1}	900	
